# Pregnancy in papillary thyroid cancer survivors

**DOI:** 10.4274/jtgga.2017.0057

**Published:** 2018-06-04

**Authors:** Kemal Beksaç, Fatih Aktoz, Gökçen Örgül, Hasan Tolga Çelik, A. Seval Özgü-Erdinç, M. Sinan Beksaç

**Affiliations:** 1 Clinic of General Surgery, University of Health Sciences, Dr. Abdurrahman Yurtaslan Ankara Oncology Training and Research Hospital, Ankara, Turkey; 2 Division of Perinatology, Department of Obstetrics and Gynecology, Hacettepe University School of Medicine, Ankara, Turkey; 3 Division of Neonatology, Department of Pediatrics, Hacettepe University School of Medicine, Ankara, Turkey; 4 Deparment of Reproductive Endocrinology, University of Health Sciences, Dr. Zekai Tahir Burak Zekai Tahir Burak Women Health Health Practice and Research Center, Ankara, Turkey; 5 Division of Neonatology, Department of Pediatrics, Hacettepe University School of Medicine, Ankara, Turkey; 6 Deparment of Reproductive Endocrinology, University of Health Sciences, Dr. Zekai Tahir Burak Zekai Tahir Burak Women Health Health Practice and Research Center, Ankara, Turkey

**Keywords:** Pregnancy, papillary thyroid cancer, thyroid cancer survivor, thyroid

## Abstract

**Objective:**

To evaluate “papillary thyroid carcinoma-pregnancy” interaction among cancer survivors.

**Material and Methods:**

The clinical records of 8 pregnant women who received treatment for papillary thyroid cancer before their pregnancy were evaluated. Clinical features, pregnancy/perinatal outcomes and high-risk factors were compared with 45 controls who were randomly assigned from the institutional perinatal medicine database.

**Results:**

Patients in the cancer group were older than the control group (34.3 vs 29.8 years). The cesarean section rate was higher (62.5% vs 33.3%) and the APGAR scores at the 1st and 5th minutes were lower in the cancer group.

**Conclusion:**

Management of pregnancies with papillary thyroid cancer treatment and follow-up requires a multidisciplinary approach with careful antenatal care and perinatal surveillance. Patients who have received papillary thyroid cancer treatment can safely undergo pregnancy.

## Introduction

It has been reported that the incidence of papillary thyroid carcinoma (PTC) has been increasing over the last decades (1,2,3). It has also been reported that differentiated thyroid cancer is more common in women and is the second most common cancer diagnosed during pregnancy and the postpartum period with a prevalence of 14 per 100.000 live births (4,5,6). However, there are conflicting results and opinions in the literature related to PTC-pregnancy interaction and optimal timing of surgical and medical intervention (7,8,9,10,11,12,13). 

Another critical issue of the “PTC-pregnancy interaction” is the management of pre- and post-operative thyroid gland problems (14,15,16). Treatment of hyperthyroidism is important in order to avoid fetal hypothyroidism (14,15). However, drug selection is also important because there is an increased risk of birth defects among methimazole users (14,16). Propylthiouracil, which is relatively hepatotoxic, is preferred in early pregnancy because of the possible teratogenicity of methimazole (14). On the other hand, management of hypothyroidism is critical in order to reduce the incidence rate of miscarriage and to maintain normal fetal brain development (14). The effects of radioactive iodine (RAI or I-131) therapy on gonads and pregnancy outcomes must also be carefully considered in patients with PTC (12,13). 

Recently, it has been reported that high levels of pro- and anti-angiogenic factors may be a risk factor for adverse outcomes via their effect on maternal thyroid function (17). Ectopic production of β human chorionic gonadotrophin (hCG) by PTC cells must also be considered in clinical practice (18). Thus, PTC and PTC-related changes may be critical both from the maternal and the perinatal morbidity/mortality rate point of view. In this clinical report, we analyzed medical/obstetric histories and the clinical features of 8 pregnancies with PTC.

## Material and Methods

Our institution’s pregnancy-associated cancer database, which consisted of 110 patients whose cancer was diagnosed between 2002 and 2015, was retrospectively evaluated (19). Eight PTC survivors were found to be eligible for the study. All of the patients received bilateral total thyroidectomy and were given RAI therapy after surgery as indicated (20). 

Clinical features, treatment modalities and pregnancy/perinatal outcomes were evaluated and compared with 45 patients as a control group who were randomly assigned from the institutional perinatal medicine database, which included all pregnancies followed in the clinic. The evaluated parameters included patients’ age, obstetric history, mode of delivery, gestational week (day) at delivery, birthweight, APGAR scores at the 1^st^, 5^th^, and 10^th^ minute, and the Beksaç et al. (21,22) obstetrics index (BOI). The BOI is an index for the assessment of risk levels of high-risk pregnancy groups, which is (number of living children + π/10)/Gravida. BOIp is the calculation of the index during the course of the last pregnancy (the perinatal outcome of the last pregnancy is not considered during the calculation).

### Statistical analysis

The Statistical Package for Social Sciences version 17 (IBM SPSS Statistics, USA) was used for data analysis. Pearson’s chi-square and Fisher’s exact tests were used for categorical variables, and the Mann-Whitney U and t-test was used for continuous variables. The last pregnancy of each patient was considered for evaluation. Sample size calculations were performed using G*Power v3.15 general power analysis program. We used 0.5 effect size, 0.8 power, and 5% level of significance (alpha) for calculations (23).

## Results

Demographics and clinical features of each group are given in Table 1. Table 2 shows obstetric history, BOI, gestational day of delivery, pregnancy outcome, birthweight of the fetus, and obstetric complications of the last pregnancy of each patient. All patients received thyroid hormone replacement therapy after their respective surgery/management (gestational and teratologic risks of the drugs were considered in all cases with necessary precautions). All patients were alive and disease free at the time of retrospective evaluation. All patients became pregnant within the first year after RAI therapy. 

The mean age in the thyroid cancer group was 34.3 years, which was statistically significantly higher than the control group, which was 29.8 years (p=0.013). We found that the cesarean section rate was higher in the thyroid cancer group (p=0.054,) which was 62.5% of the cases. We also found that the 1-minute and 5-minute APGAR scores were statistically significantly lower in the cancer group (p=0.022 and p=0.03, respectively). The mean interval between cancer treatment and pregnancy was 4.5±3.11 years (range, 1-11 years).

In this clinical series, 7 of 8 patients delivered (five by cesarean section and two by vaginal delivery), and one pregnancy ended with spontaneous abortion. One neonate had corpus callosum agenesis (the family refused to have induced abortion) and was delivered vaginally. Neonates of a twin pregnancy were delivered by cesarean section at the 37^th^ gestational week (in vitro fertilization twin; 2060 and 2060 g male neonates) with no complications. One patient was preeclamptic and delivered a 1900 g female neonate at the 36^th^ gestational week (this neonate was discharged from the intensive neonatal care unit with no complications). 

There was no statistically significant difference between the control and study groups in terms of birthweight (3195±566 g and 3051±886 g, respectively), and the gestational week at delivery was lower in patients with cancer (266 days vs 271 days; p=0.016). We believe that this finding is not critical in clinical practice. The obstetric history and perinatal outcomes of previous pregnancies of both groups were evaluated using BOIp and no statistical differences were observed.

## Discussion

Thyroid cancer, with an incidence of 9 per 100.000 persons per year (24), has seriously increased over the last two decades, mainly due to the papillary type (1,2). It has also been reported that differentiated thyroid cancer is more common in women and is the second most common cancer diagnosed during pregnancy and the postpartum period with a prevalence of 14 per 100,000 live births (4,5,6). Several studies have suggested an association between PTC and reproductive variables such as estrogen and hCG (2,6). 

The “Pregnancy-PTC” interaction is full of controversies. hCG, which is a weak thyroid-stimulating hormone agonist, may sometimes be secreted by PTC cells (18,25). On the other hand, various growth factors and pro- and anti-angiogenic factors may influence perinatal outcome via thyroid (dys) function (17). Some authors reported that pregnancy had no effect on PTC, whereas others suggested that PTC may progress during pregnancy (9,10). However, there is a consensus about the timing of surgery when PTC is diagnosed during pregnancy (7,8,26). Surgery can be delayed until after delivery in appropriate patients. It has been reported that I-131 therapy may result in transient ovarian dysfunction, but subsequent pregnancies are safe without any significant consequence to perinatal outcome (12,13). In our study, we have shown that pregnant women with thyroid cancer history are older than the general population. Actually, this finding is not surprising because most women should be in remission or disease free before getting pregnant for better maternal and fetal outcomes. The long treatment period and awareness the fetal teratogenic effect of medication seems to force women to delay their pregnancies. We believe that pregnancy planning should be postponed until after having proper PTC treatment due to these uncertainties.

Cancer-related worry is very important for patients with PTC who want to become pregnant (27). On the other hand, stress is an important determinant for patients whose PTC diagnosis was made during pregnancy (28). Waiting for surgery is another problem to be managed during pregnancy due to long wait times (29). Our findings demonstrate that PTC survivors might be encouraged to become pregnant if they are willing to do so even when considering the facts mentioned above.

The effect of PTC on pregnancy needs to be studied. There are no case-control prospective studies on the effect of PTC on obstetrics/perinatal complications. In our case series, we demonstrated that 1-minute and 5-minute APGAR scores were lower in PTC survivors and the cesarean section rate is higher in this group of patients compared with the control group. PTC-related immune and metabolic changes may be responsible for the inflammatory changes at the materno-fetal interface (injury of the cellular components of the intervillous space) and this might be the reason of impaired fetal perfusion going together with stress-intolerant babies, increased cesarean section rates, and low APGAR scores.

All these factors and patient-specific surgical/medical treatment modalities necessitate a patient-specific antenatal care program and careful perinatal surveillance.

## Figures and Tables

**Table 1:**
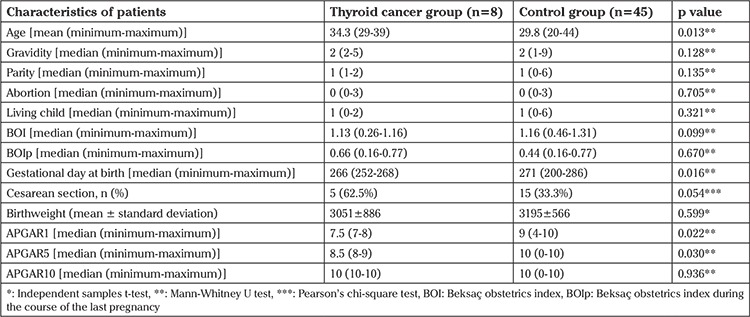
Demographic and clinical features of patients

**Table 2:**
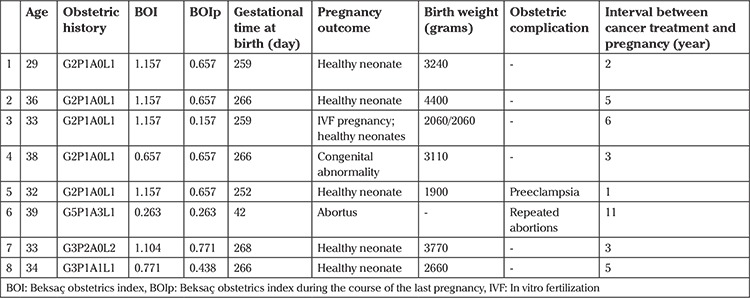
Pregnancy outcome variables of patients with thyroid cancer
